# The matching relationship and driving mechanism of elderly medical care resources and elderly population in China: A study based on provincial perspective

**DOI:** 10.1097/MD.0000000000040882

**Published:** 2024-12-13

**Authors:** Zhongli Qiao, Ye Ding, Yu Zhu, Shangren Qin

**Affiliations:** a School of Public Health, Hangzhou Normal University, Hangzhou, Zhejiang, China; b School of Public Health, Hangzhou Medical College, Hangzhou, Zhejiang, China.

**Keywords:** China, elderly population, matching degree, medical care beds for the elderly, medical resource

## Abstract

With the exacerbation of population aging in China, policies have been formulated to provide elderly medical care resources. However, research on the matching situation of these resources with the elderly population (EP) is relatively scarce. This study collected data on elderly medical care resources and the EP from various provinces in mainland China from 2011 to 2017. Using Spearman analysis, the correlation between elderly medical care resources and the EP was explored. Based on geographical concentration, the resource and population matching conditions of each province were calculated and categorized, while the deviation across the nation was measured. Finally, panel regression was used to identify driving factors affecting provincial matching degrees. From 2011 to 2017, the EP aged 60 or above in China grew from 173 million to 235 million, an increase of 35.84%. In contrast, the number of elderly medical care beds grew from 118,000 in 2011 to 135,000 in 2017, an increase of only 14.41%. Although a positive correlation has emerged between China’s elderly medical care resources and the EP in recent years, the deviation between the two has been expanding annually. Among all provinces, more than one-third still lag behind in terms of elderly medical care resources. Moreover, the matching degree is closely related to economic levels, with significant differences observed between the eastern and western regions—the developed eastern regions have higher matching rates, while the less developed regions experience lower rates. The driving factors influencing provincial matching degrees have been identified as per capita GDP (β = 0.67, *P* = .010) and fiscal health expenditure (β = 0.22, *P* < .001). The matching degree between elderly medical care resources and the EP in China urgently needs to be improved. Economic conditions have a significant impact on the matching degree. To better serve the EP, it is needed to increase investments in elderly medical care resources in the western and northeastern regions, and promote an increase in the matching degree by enhancing per capita gross domestic product and fiscal health expenditure.

## 1. Introduction

The issue of population aging in China is increasingly prominent. According to statistics, by the end of 2021, the population aged 65 and above approached 190 million, accounting for 13.5% of the total population.^[1]^ With the ongoing trend of aging, the burden on the existing medical system grows heavier.^[2]^ Projections indicate that by 2030, the elderly population (EP) suffering from at least one or more chronic diseases will increase by at least 40% compared to 2020.^[3]^

In view of the rapid process of aging, China has implemented numerous supporting policies to ensure an adequate supply of elderly medical care resources, such as the launch of the “Ten-Year Medical Reform” project, the implementation of the “Healthy China 2030” action plan, and the establishment of the National Center for Clinical Research on Geriatric Diseases.^[2]^ Although the Chinese government has made great efforts, whether the nationwide matching condition of elderly medical care resources and the EP is satisfactory remains a subject for further discussion.

Therefore, this study aims to answer the following 3 main questions: 1. Can elderly medical care resources and the EP in China and its various provinces match each other? 2. Is there a significant difference in the matching situation between provinces? 3. What are the driving factors affecting the matching degree of elderly medical care resources and the EP? Understanding the matching degree and its driving mechanism between China’s elderly medical care resources and the EP not only helps accurately identify the shortcomings in the allocation of elderly medical care resources but also provides forward-looking reference for decisions regarding local health funding input and allocation of elderly medical care resources.

This study first analyzes how medical care resources and the EP influence each other and proposes empirical research hypotheses. It then elaborates on the methodological framework adopted for the research hypotheses. After presenting the research results, it interprets the empirical findings. The final section includes discussion and conclusion, clarifying key discoveries and proposing policy recommendations for improving the matching degree.

## 2. Literature review and research hypothesis

### 2.1. Mutual impact of medical care resources and the EP

Firstly, medical resources have an impact on the EP. Appropriate allocation of medical resources (such as hospital beds and county-level hospitals) can effectively reduce the medical expenses of the elderly.^[4]^ Related literature further explores the accessibility of medical resources for the elderly and finds that there is imbalance and inequity in their access to medical services—this inequity increases with distance from the city center to peripheral areas.^[5]^ Some studies emphasize that successful management of aging challenges requires a combination of active social support and a well-structured healthcare system, aiming to achieve comprehensive considerations of medical resource supply and equitable access.^[6,7]^ Secondly, the EP has an impact on medical resources. Hospital diagnosis statistics from the German Federal Statistical Office show that changes in the number of the EP will reshape the configuration of hospital departments.^[8]^ The U.S. Census Bureau points out that population aging will lead to an annual increase of approximately 0.74% in the use of medical services.^[9]^ Hong Kong scholars, based on discharge data from 2001 to 2012, argue that the rapid growth of the EP has resulted in an increasingly severe shortage of medical resources in Hong Kong.^[10]^ Additionally, exploration of the evolution of China’s health resource geographical pattern shows that key factors influencing the spatial distribution of provincial health resources include children and the EP, government input into healthcare, and added value of the service industry.^[11]^ It has been found that as the proportion of the EP rises, the number of beds in hospitals and health centers also increases accordingly, indicating a positive correlation between the two.^[12,13]^

Hypothesis 1: There is a positive correlation between elderly medical care resources and the EP.

### 2.2. Matching degree of medical care resources and the EP

Although many studies have pointed out the correlation between medical care resources and the EP, research specifically exploring their spatial matching condition is relatively rare. A study in Taiwan calculated the spatial matching degree of local elderly community care resources and the EP in 2017 and found that the distribution of the two was inconsistent.^[14]^ Another study shows that the spatio-temporal matching degree between the aging rate and the number of medical resources per thousand residents in mainland China is generally low, with the spatial matching degree lower where the aging rate is higher.^[15]^ It should be noted that these studies mainly focus on “all medical care resources at the macro level” rather than specifically on “medical care resources only for the elderly.” This paper will focus on the matching degree between “medical care resources only for the elderly” and EP.

Hypothesis 2: The match between elderly medical care resources and EP in China is relatively low, with significant regional disparities in matching levels across provinces.

### 2.3. Drivers of the match between medical care resource and EP

Chinese scholars have found that the discrepancy in the match between elderly care resources and the EP across regions is mainly determined by the changes in the share of elderly care resources, while changes in EP share play a secondary role. That is, the aggregation of elderly care resources in each region exceeds that of EP. Hence, the allocation mechanism of elderly care resources should be guided by the base number of EP in the future.^[16]^ This paper will attempt to explore the dominant and subordinate factors of the match between elderly medical care resources and EP.

In addition, research has found that due to economic differences, there are disparities in the match between medical care resources for the elderly and EP in different provinces of China. Provinces with higher levels of economic development, such as those in the east, have a higher degree of match, whereas provinces with relatively backward economic development in the central and western parts have a lower degree of match.^[15,16]^ This suggests that the level of economic development may be an important driving factor affecting the match.

First, population aging can promote economic growth by increasing the expenditure on pensions and health costs. China’s aging index significantly influences both per capita medical expenditure and per capita GDP.^[17]^ In areas with a high level of economic development, population aging will drive increases in social security, employment, and healthcare consumption. Conversely, the growth in healthcare consumption can positively stimulate economic development.^[18]^ Therefore, relevant studies indicate that by increasing pension insurance expenditure and healthcare consumption, population aging can play a positive role in economic growth.^[19]^

Second, economic growth will drive the expansion of healthcare service demand and the improvement of healthcare resource allocation. Research shows that the level of economic development (e.g., GDP) in Chinese provinces, urbanization wages, and the level of fiscal health expenditure are significantly positively correlated with healthcare resource aggregation capability.^[20,21]^ Moreover, an increase in per capita GDP helps improve the efficiency of healthcare services.^[22]^ In other words, an improvement in economic level aids in increasing the allocation of medical care resources, making it better match the health needs of the EP.

Hypothesis 3: The change in the match between elderly medical care resources and EP in China is mainly driven by elderly medical care resources, with EP in a subordinate position.

Hypothesis 4: The primary positive drivers of their matching degree are economic factors, such as per capita GDP and fiscal health expenditure.

## 3. Research methodology

### 3.1. Data definitions

In this study, the term “EP” specifically refers to individuals aged 60 and above. Although “elderly medical care resources” should directly point to medical care resources provided for the elderly, this type of resource cannot fully cover all medical care resources at the macro level, and its data collection process is quite complex. Therefore, this research chooses to use “NEMCB” as a representative indicator.

Additionally, this study also explores potential driving factors that affect the matching of allocation. Firstly, based on existing literature reports, economic factors such as per capita GDP and fiscal health expenditure have been confirmed as the leading forces in the allocation of medical resources.^[20,21]^ Therefore, this research considers per capita GDP, fiscal health expenditure, percentage of health expenditure in GDP, and per capita total health expenditure as key driving factors of the study. Secondly, drawing from related studies,^[19]^ 2 adjustment variables, urbanization rate and natural population growth rate, are also included in the model. Lastly, given that healthcare security (such as medical insurance) has a profound influence on the distribution of medical resources,^[23]^ the core indicator of healthcare security, number of urban residents and employees insured by basic medical insurance, is also taken into account as a potential driving force.

### 3.2. Data sources

This study relies on data from various provinces in China (excluding Taiwan, Hong Kong, and Macao) from 2011 to 2017. Particularly, data related to the number of EP aged 60 and above and number of elderly medical care beds (NEMCB) were directly quoted from the China Civil Affairs Statistical Yearbook. However, it should be noted that relevant data for Tibet and Qinghai Provinces have not been fully collected, and there are some missing data for Hainan and Chongqing Provinces (e.g., data for Hainan is missing from 2011 to 2014, and data for Chongqing is missing from 2013 to 2014). Other indicators like per capita GDP, fiscal health expenditure, percentage of health expenditure in GDP, per capita total health expenditure, number of urban residents and employees insured by basic medical insurance were directly quoted from the China Health and Family Planning Statistical Yearbook. Simultaneously, data on the urbanization rate and natural population growth rate are sourced from the China Statistical Yearbook. Given the limitations of data completeness, this research analyzes data from various provinces in China from 2011 to 2017.

Following the rules published by the National Bureau of Statistics of China,^[24]^ this research divides mainland China into 4 regions—the East, Central, West, and Northeast—for study, based on their respective levels of socioeconomic development.

### 3.3. Data analysis method

#### 3.3.1. Analyzing the correlation between elderly medical care resources and EP

This study initially calculates the national share of elderly medical care resources and EP for each province annually (NEMCB% and EP%). Then, Spearman correlation analysis is used to examine whether there is a correlation between them. Based on this, scatter plots are drawn for each year’s data (with the *y*-axis representing the share of elderly medical care resources and the *x*-axis representing the share of EP) to observe their trends visually, and corresponding regression equations are fitted to further clarify the association between the two indicators.

#### 3.3.2. Calculating the provincial matching degree (RI) between elderly medical care resources and EP

Once the correlation between elderly medical care resources and EP is established, the geographical concentration-based match between the elderly medical care resources and EP for each province (RI), as well as the national deviation (M), is calculated. This spatial matching method has been widely applied in various fields, such as the match between economy and population,^[25]^ elderly care resources and EP,^[16]^ and its maturity has been recognized.

Specifically, this study will calculate the concentration of elderly medical care resources $R_{med_{i}}$ and the concentration of EP $R_{pop_{i}}$ for each province annually, as indicated in Eq. (1).


$$\begin{array}{l} R_{med_{i}}=\frac{med_{i}/\sum med_{i}}{ter_{i}/\sum ter_{i}}\;\;\;\;\;\;\;\;\;\;\;\;\;\;\;\;\;\;\;\;\;\;\;\;\;\;\;\;\;R_{pop_{i}}=\frac{pop_{i}/\sum pop_{i}}{ter_{i}/\sum ter_{i}}\;\;\; \end{array}$$ (1)

In Eq. (1), $pop_{i}$ and $med_{i}$ represent the number of EP and the volume of elderly medical care resources in province *i* during a specific period, respectively; $ter_{i}$ represents the land area of province *i*. $\sum pop_{i}$, $\sum med_{i}$, and $\sum ter_{i}$ refer to the national number of EP, total volume of elderly medical care resources, and land area, respectively.

Based on geographical concentration, drawing on related literature, the match between elderly medical care resources and EP for each province can be further calculated annually (RI),^[25,26]^ as shown in Eq. (2).


$$\begin{array}{l} RI=\frac{R_{med_{i}}}{R_{pop_{i}}}=\frac{med_{i}/\sum med_{i}}{ter_{i}/\sum ter_{i}}÷\frac{pop_{i}/\sum pop_{i}}{ter_{i}/\sum ter_{i}}=\frac{med_{i}/\sum med_{i}}{pop_{i}/\sum pop_{i}} \end{array}$$ (2)

From Eq. (2), it can be seen that the match coefficient RI between elderly medical care resources and EP can be regarded as the ratio between the share of elderly medical care resources and the share of EP in a province. Then, according to the size of RI, the matching degree classification of each province is further divided. When RI is >1.2, the corresponding province is considered as resource-advanced, meaning that the aggregation of elderly medical care resources in this province exceeds its EP level, significantly highlighting its advantage in elderly medical care resources compared to the national average. When RI is between 0.8 and 1.2, the corresponding province is considered relatively matched, i.e., the distribution of elderly medical care resources in the area basically matches the distribution of EP. If RI is <0.8, the province is classified as resource-lagged, indicating that there is a poor match between elderly medical care resources and EP in the area, and the allocation capability of its elderly medical care resources needs to be improved. Through such classifications, a more comprehensive grasp of the differences in elderly medical care resources across various regions and provinces can be obtained.

#### 3.3.3. Calculating the national deviation (M) between elderly medical care resources and EP

Given that the matching degree RI can only describe the match between elderly medical care resources and EP in each province but cannot reflect the nationwide matching situation, this study integrates the matching degree indicators of each province and further calculates the deviation index M^[25]^ to describe the annual national match between elderly medical care resources and EP, as shown in Eq. (3).

$$\begin{array}{l} M=\sqrt{\frac{\sum \frac{\sqrt{2}}{2}{\left(\frac{med_{i}}{\sum med_{i}}-\frac{pop_{i}}{\sum pop_{i}}\right)}^{2}}{n}} \end{array}$$ (3)

In Eq. (3), *n* represents the number of provinces. The deviation index M reveals the overall matching relationship between elderly medical care resources and EP nationwide. The larger the deviation index value, the higher the divergence of the overall distribution of elderly medical care resources and EP nationwide, indicating a greater mismatch between the two. Conversely, if the deviation index value is small, it indicates a better match between the two distributions.

#### 3.3.4. Analyzing dominant factors and influences on the match between elderly medical care resources and EP

To further propose targeted improvement measures, this study also analyzes the dominant factors and driving forces affecting the match between elderly medical care resources and EP.

First, the annual share of elderly medical care resources in the national total, the share of EP in the national total, and the regional matching degree for each region (East, Central, West, and Northeast) are calculated. Then, the aforementioned results are plotted over time by region. By observing these trends, the dominant factors influencing changes in the Matching Degree can be identified. For instance, if the trend of the matching degree change resembles that of the changes in elderly medical care resources but differs from that of EP, it can be determined that elderly medical care resources are the dominant factor influencing Matching Degree changes.

Next, using the annual match degree of each province as the dependent variable, and per capita GDP, fiscal health expenditure, percentage of health expenditure in GDP, per capita total health expenditure, number of urban residents and employees insured by basic medical insurance, urbanization rate, and natural population growth rate as independent variables, a panel data regression model is used to analyze the driving factors influencing the match between elderly medical care resources and EP.

Prior to performing panel data regression, this study checks for multicollinearity among the independent variables. The results show that all variables have a variance inflation factor <10, indicating no issue of multicollinearity among the independent variables. To eliminate the influence of dimensions, some independent variables (such as per capita GDP, fiscal health expenditure, per capita total health expenditure, number of urban residents and employees insured by basic medical insurance) are logged before incorporated into the regression model. Concurrently, to control the changes in the macro environment (e.g., economic conditions each year), dummy variables for the year are added to the model. In addition, a unit root test is conducted on each independent variable before regression. The results show that nonstationary variables (*P* > .05) need to be first-differenced before included in the model. Finally, the type of model and the determination of regression coefficients are made based on the results of the Hausman test (a random-effects model is chosen if *P* > .05, a fixed-effect model if *P* < .05).

All statistical tests are 2-sided, with differences deemed statistically significant when *P* values are <.05. All data statistical analyses were performed using STATA 17.0 software (STATA Corp, College Station, TX).

## 4. Research results

This study collected data from 29 provinces in mainland China (excluding Tibet and Qinghai) during 2011 to 2017. During this period, the EP aged over 60 in China increased from 173 million to 235 million, an increase of 35.84%. Meanwhile, NEMCB also increased yearly, from 118,000 in 2011 to 135,000 in 2017, but the growth rate was only 14.41%.

### 4.1. Correlation between elderly medical care resources and EP

**Figure 1.**
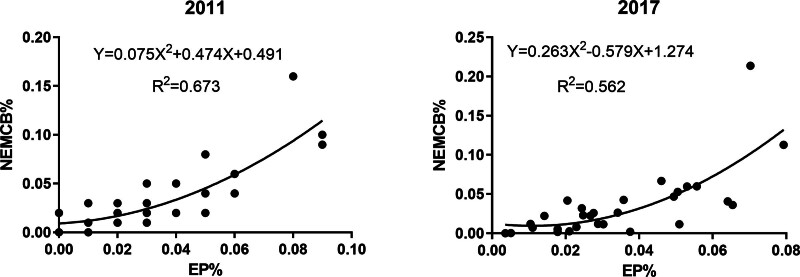
Trend changes of elderly medical care resources and EP. EP = elderly population.

Through Spearman correlation analysis (Table 1), it can be found that there is a positive correlation between the share of elderly medical care resources and the share of EP in each province from 2011 to 2017. The correlation peaked in 2014 and then slightly declined. Similarly, the trend of scatter plot fitting (Fig. 1) also shows that the share of elderly medical care resources increases with the growth of EP share, although the growth trend in 2017 is somewhat slower than that in 2011.

**Table 1:** Correlation between NEMCB% and EP%.

	2011	2012	2013	2014	2015	2016	2017
Spearman correlation coefficient	0.770	0.846	0.862	0.887	0.775	0.764	0.739
*P*-value	<.001	<.001	<.001	<.001	<.001	<.001	<.001

EP = elderly population, NEMCB = number of elderly medical care beds.

These results all indicate a positive correlation between the spatial distribution of elderly medical care resources and EP, that is, the more concentrated the EP, the more concentrated the elderly medical care resources, suggesting a certain match between the two.

### 4.2. Provincial matching of elderly medical care resources and EP

Based on geographic concentration, the spatial matching index (RI) between elderly medical care resources and EP for each province was calculated, and provinces were divided into 3 categories according to the size of the RI: resource-leading (RI > 1.2), relatively-matching (0.8 ≤ RI ≤ 1.2), and resource-lagging (RI < 0.8).

On a national level, the number of resource-leading provinces in China decreased from 10 (accounting for 35.71%) in 2011 to 5 (17.86%) in 2012, then remained relatively stable at about 6 (approximately 20.69%) over the next 5 years. The relatively-matching provinces fluctuated significantly in the first 2 years but stabilized at around 12 (about 41.38%) in the subsequent 5 years. The number of resource-lagging provinces remained fairly consistent at about 11 (approximately 37.93%). Overall, a significant proportion of provinces are still resource-lagging. The matching condition between the elderly medical care resources and EP in each province needs further improvement.

**Figure 2.**
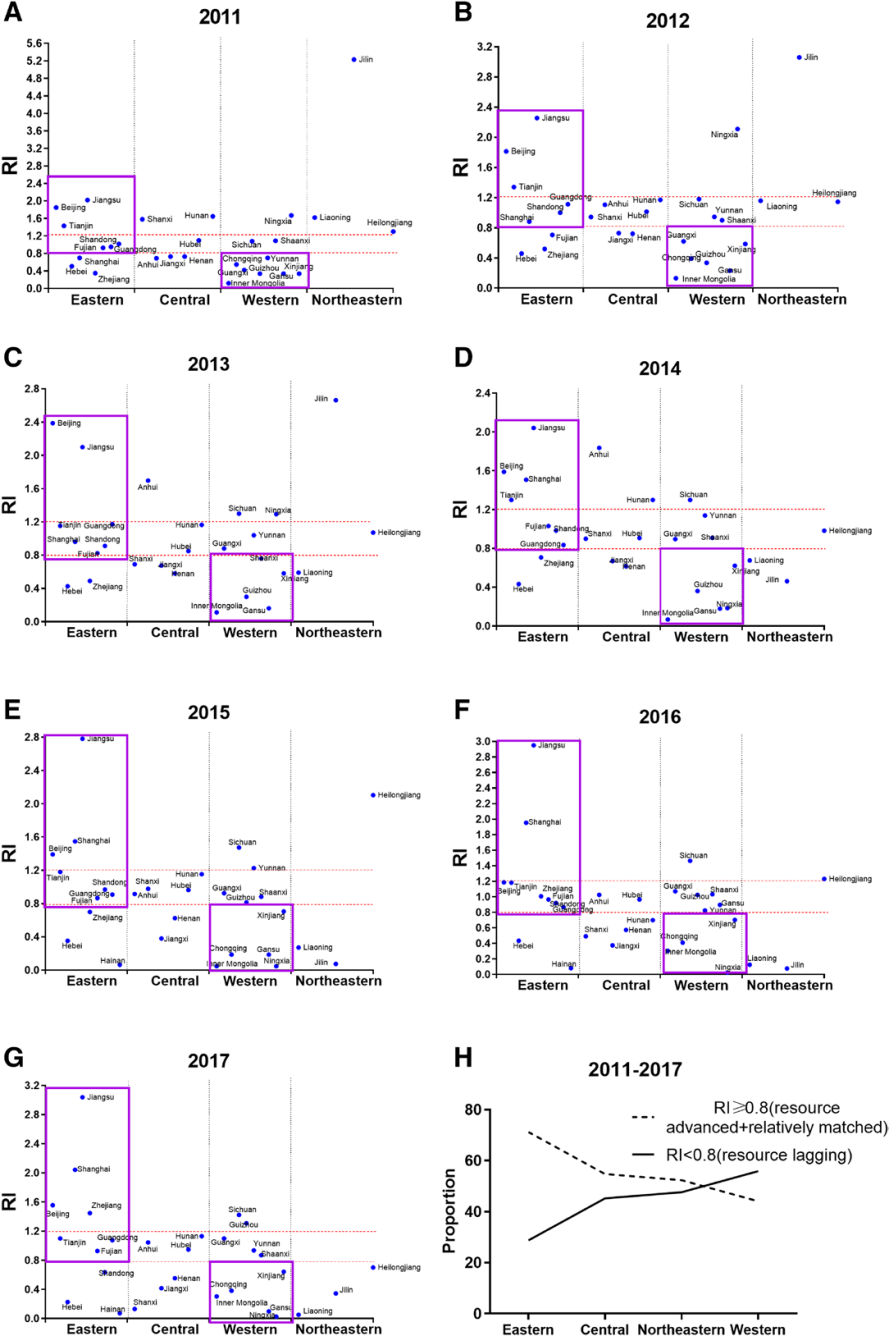
Matching degree of elderly medical care resources and EP in Chinese provinces (provincial level). EP = elderly population.

Regionally, the Eastern area, the most economically developed region, has the highest proportion of resource-leading provinces and the lowest proportion of resource-lagging ones. In contrast, the Western area, as the least economically developed region, has a higher number of resource-lagging provinces (Fig. 2), with a proportion exceeding other areas every year (over 50% consistently). Besides, the Northeast region witnesses a noticeable decline in matching degree, with all its 3 provinces falling from resource-leading in 2011 to resource-lagging in 2017, which may be related to its recent economic slowdown. Therefore, there is a correlation between the economic level and the matching degree: regions with better economic conditions have higher matching degrees and vice versa.

### 4.3. National matching of elderly medical care resources and EP

Since the RI can only describe the matching condition between elderly medical care resources and EP for each province, it does not reflect the overall national situation. Therefore, the deviation index (M) was calculated as a comprehensive tool to describe the annual matching condition of elderly medical care resources and EP nationwide. The larger the deviation index, the poorer the matching degree.

**Figure 3.**
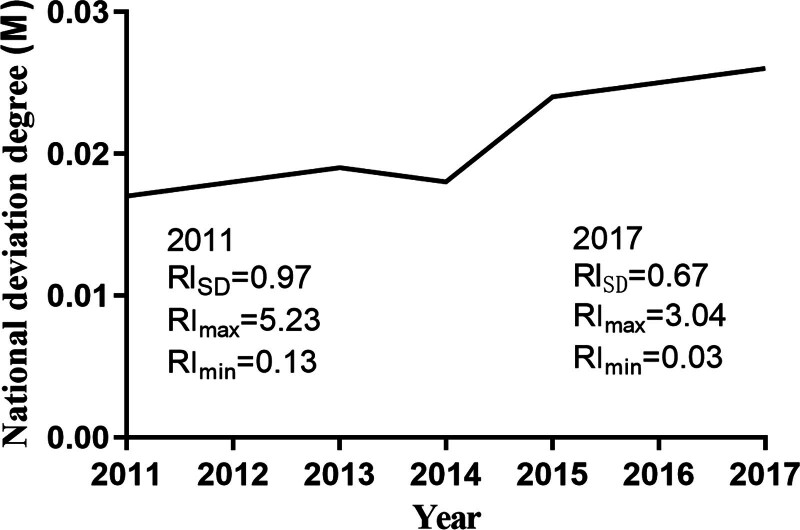
Overall matching degree between elderly medical care resources and EP in China (national level). EP = elderly population.

The results (Fig. 3) show that the national deviation index fell slightly in 2014 but then rose sharply. Overall, the deviation index is on an upward trend, increasing from 0.017 in 2011 to 0.026 in 2017, an increase of 52.94%. This indicates that the matching condition of elderly medical care resources and EP nationwide is not optimistic, with the agglomeration of elderly medical care resources deviating more and more from that of EP.

### 4.4. Dominant factors and driving forces of the match between elderly medical care resources and EP

**Figure 4.**
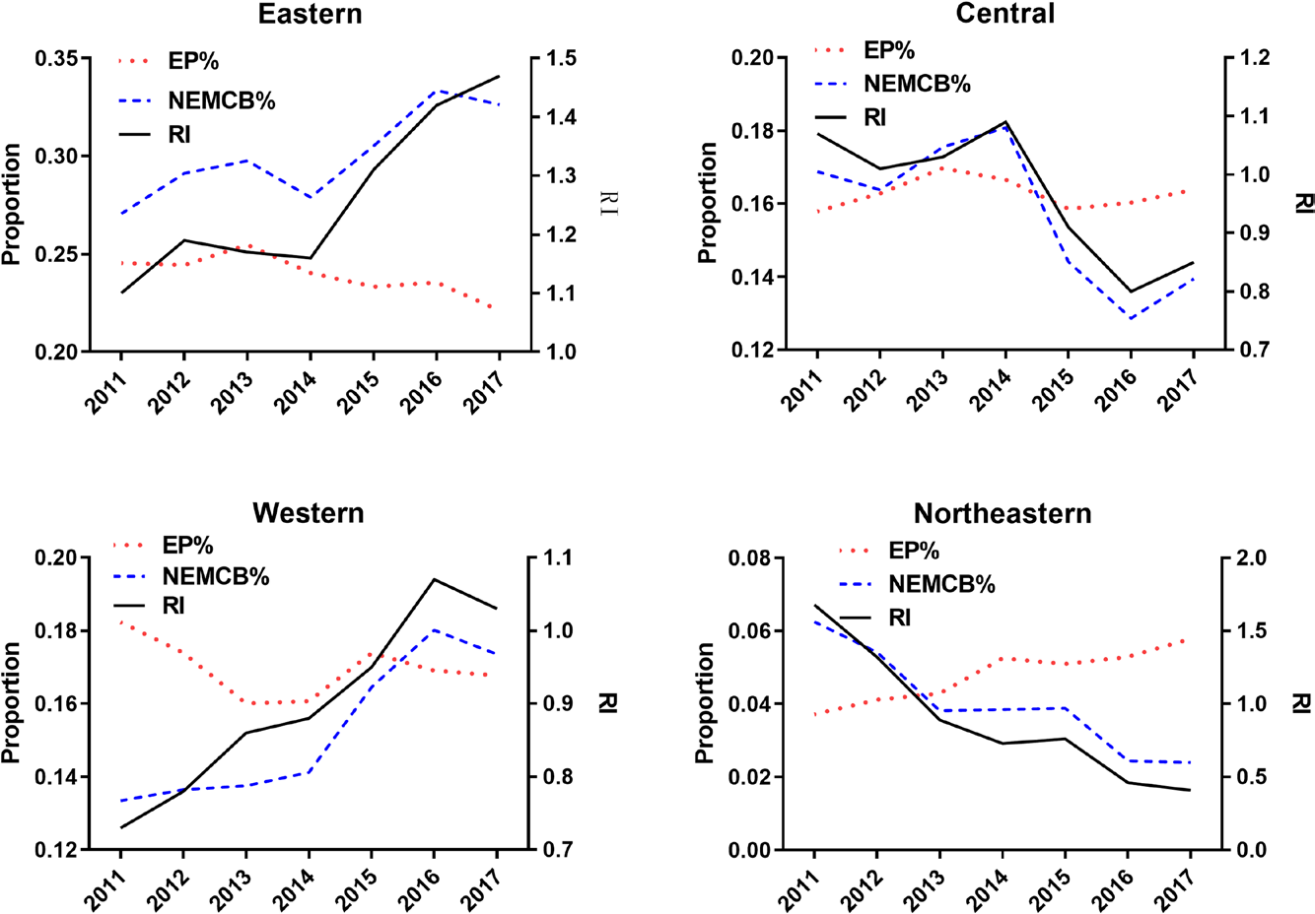
Changes in elderly medical care resources, EP, and their matching degree in China (study on dominant factors). EP = elderly population.

By describing the temporal trends of the share of elderly medical care resources, EP share, and regional matching degree (Fig. 4), it can be inferred that the dominant factor influencing the matching degree is the elderly medical care resources, as its changing trend is similar to that of the matching degree, but different from the EP’s.

Furthermore, the driving forces behind the matching degree were also analyzed. As the *P*-value of the Hausman test was >.05 (*P* = .797), a random-effects model was used for regression fitting (Table 2). Among the independent variables, per capita GDP and fiscal health expenditure coefficients were positive, and their *P*-values were <.05. This suggests that per capita GDP and fiscal health expenditure are driving factors affecting the matching degree, and their increase contributes to improving the match between elderly medical care resources and EP.

**Table 2:** Influencing factors of matching degree between NEMCB and EP.

Factors	Coefficient (95% CI)	*P*-value
Natural population growth rate	0.01 (‐0.04 to 0.06)	.746
Urbanization rate	‐0.05 (‐0.30 to 0.20)	.694
Per capita GDP	0.67 (0.16 to 1.17)*	.010
Fiscal health expenditure	0.22 (0.14 to 0.30)***	<.001
Percentage of health expenditure in GDP	0.03 (‐0.18 to 0.23)	.802
Per capita total health expenditure	‐0.02 (‐0.04 to 0.00)	.110
Number of urban residents and employees insured by basic medical insurance	‐0.06 (‐0.39 to 0.28)	.745

EP = elderly population, GDP = gross domestic product, NEMCB = number of elderly medical care beds.

*R*^2^ = 0.248.

* *P* < .05.

*** *P* < .001.

## 5. Discussion

This study describes the status of elderly medical care resources and EP in various provinces of China from 2011 to 2017, and discusses their matching conditions and influencing factors.

China has seen a rapid growth in EP in recent years, facing significant aging challenges.^[27]^ With the increase of EP, the proportion of chronic diseases in disease burden is also increasing.^[28]^ This requires the government to expand the allocation of elderly medical care resources in the healthcare sector to cope with the challenges brought by aging. Although this study confirms a positive correlation between China’s elderly medical care resources and EP, the growth rate of elderly medical care resources is significantly lower than that of EP.

This study further explores the matching condition between elderly medical care resources and EP at the provincial level. The results show that over one-third of provinces in China are still resource-lagging, meaning that the agglomeration of elderly medical care resources in these provinces lags behind the local agglomeration of EP. The economically developed eastern region has a better matching degree, with more resource-leading provinces. While in the less developed western region, the matching degree is poor, with more resource-lagging provinces. This result is consistent with existing studies.^[15]^ In China, the concentration and balance of medical resources are superior in the east compared to the west,^[20,21]^ so the growth speed of medical resources in the eastern region can keep up with population growth, thereby improving their matching degree. Furthermore, it was found that all 3 provinces in the Northeast region shifted from resource-leading in 2011 to resource-lagging in 2017, which may be related to the recent economic slowdown in the Northeast.^[29]^ Therefore, it is inferred that there might be a relationship between the economic level and the matching degree, that is, the matching degree might change accordingly with changes in the economic level.

In addition to discussing the matching degree at the provincial level, this study also calculates the deviation between elderly medical care resources and EP nationwide. The results show that the deviation is increasing year by year in China, indicating that the allocation of elderly medical care resources is increasingly deviating from the growth of EP. Overall, there are still severe inequalities in the allocation of medical resources in China (such as uneven distribution between east and west),^[30]^ and there is a lot of room for improvement in terms of quantity. For example, the allocation of public health resources has not yet met the requirements of China’s national medical and health service system plan.^[31]^ Therefore, there are both distribution and quantity problems in the allocation of elderly medical care resources nationwide, which lead it to increasingly deviate from the development trend of national EP. This also validates another finding: the change in the matching degree between elderly medical care resources and EP is mainly driven by the changes in elderly medical care resources, rather than dominated by EP factors.

Further, this study finds that per capita GDP and public health expenditure can promote the improvement of the matching degree and can be seen as driving factors of the matching degree. The possible mechanism is that with population aging, economic development can be stimulated, thereby improving the per capita GDP and public health expenditure of each province.^[17]^ Moreover, the increase in per capita GDP and public health expenditure can stimulate the improvement of health resource allocation, ultimately making health resources more closely match with EP.^[20,21]^

This study has some limitations. Firstly, this study analyzes the matching degree of elderly medical care resources and EP using provinces as observation units. However, the province is a large unit and cannot accurately measure the accessibility of EP to medical resources. That is, the matching degree can only roughly reflect the accessibility of elderly medical care resources and the level of population aging in a province. If higher matching accuracy is desired, more precise geographical latitude and longitude data (such as remote sensing data) are needed. Secondly, despite efforts to collect data, this study only uses NEMCB as an indicator of elderly medical care resources, which may limit the representativeness of research results.

## 6. Conclusion

This study finds that although a positive correlation exists between elderly medical care resources and EP in China in recent years, the growth rate of elderly medical care resources is slower compared to EP, with their deviation degree growing annually. At the provincial level, the overall matching degree between elderly medical care resources and EP is not high, with over one-third of provinces still resource-lagging. The matching degree varies with economic levels, with obvious disparities between the eastern and western regions. Moreover, the matching degree is better in economically developed eastern regions and poorer in less developed western regions. Additionally, it has been found that a province’s per capita GDP and public health expenditure are key driving factors influencing the matching degree, and the improvement of these 2 economic indicators helps to increase the matching degree.

Theoretically, this study enriches research on the impacts of aging societies and advances the theoretical system of “medical resource allocation.” In practice, gaining a comprehensive understanding of the matching degree between EP medical care resources and EP in various Chinese provinces benefits the rapid identification of deficiencies in the allocation of medical care resources for the aging population, providing a forward-looking reference for decision-making related to provincial-level health fund investment and the allocation of elderly medical care resources. For example: 1. Western and Northeastern regions are the main concentration areas of resource-lagging provinces, so the Chinese government should prioritize these 2 regions for attention and investment. 2. The economic level is closely related to the matching degree. Regions can promote the optimization of elderly medical resource allocation and make it better match with EP by increasing per capita GDP and public health expenditure. To sum up, this study holds significant implications for understanding the current status and challenges of the allocation of medical care resources for the aging population in various Chinese provinces, and provides strong evidence for related decision-making.

## Author contributions

**Conceptualization:** Shangren Qin.

**Data curation:** Zhongli Qiao, Ye Ding.

**Formal analysis:** Zhongli Qiao, Ye Ding.

**Funding acquisition:** Shangren Qin.

**Methodology:** Shangren Qin.

**Project administration:** Shangren Qin.

**Resources:** Zhongli Qiao, Ye Ding.

**Software:** Zhongli Qiao, Ye Ding.

**Supervision:** Shangren Qin.

**Writing – original draft:** Zhongli Qiao, Ye Ding.

**Writing – review & editing:** Yu Zhu, Shangren Qin.
